# Legionella pneumonia: A Case of Fever Prolonged for 10 Days

**DOI:** 10.7759/cureus.59979

**Published:** 2024-05-09

**Authors:** Matthew W Spretz, Martin M Orrukem, Bisma Khwaja, Bernard Karnath

**Affiliations:** 1 Internal Medicine, University of Texas Medical Branch, Galveston, USA

**Keywords:** legionella, acute kidney injury, elevated creatinine, pneumonia, urine antigen test, ct chest, fluoroquinolone, legionnaires disease, legionella pneumonia, atypical pneumonia

## Abstract

Legionnaires’ disease is a potentially severe type of pneumonia most often caused by the organism *Legionella pneumophila*. Exposure to this bacterial pathogen typically happens in the community but may also occur in the hospital setting. This report describes the case of a patient who presented due to 10 days of fever, shortness of breath, and diarrhea, with initial imaging demonstrating multifocal pneumonia. The patient was appropriately started on empiric antibiotics for community-acquired pneumonia and admitted to the medicine floor. The patient showed no meaningful improvement in his initial hospital course on empiric antibiotics with continued oxygen requirements. Meanwhile, urine Legionella antigen testing returned positive on hospital day four, and after tailoring antibiotics accordingly, the patient's clinical status improved significantly. This case report highlights the efficacy of broad testing in the initial admission and the need for constant re-evaluation in the context of a patient not improving with appropriate therapy.

## Introduction

Legionella pneumonia, commonly referred to as Legionnaires’ disease, is a severe form of pneumonia most often caused by the bacterium *Legionella pneumophila*. This potentially life-threatening illness gained notoriety after an outbreak among the attendees of the 1976 American Legion Convention in Philadelphia, hence its name. Epidemiologically, Legionella is often associated with contaminated water systems, such as those found in large buildings, hospitals, cruise ships, and, in some cases, an average air condition unit [1]. Transmission is primarily through inhalation of aerosolized water droplets containing the bacteria. Typical presentations of Legionella pneumonia may include a high fever, chills, cough (which may or may not be productive), shortness of breath, muscle aches, and headaches [2,3]. Interestingly, gastrointestinal symptoms such as nausea, vomiting, and diarrhea commonly occur [4]. Laboratory abnormalities may involve elevated aminotransferases, elevated C-reactive protein, and hyponatremia. These symptoms can rapidly progress to severe respiratory distress, making early recognition and appropriate treatment crucial management steps. Our patient had an atypical presentation in that he had a dry, nonproductive cough and displayed minimal response to empiric treatment that covered Legionella. Ultimately, the team made a diagnosis on hospital day four, with the delay primarily attributed to the sample requiring testing at an outside facility.

## Case presentation

**Figure 1:**
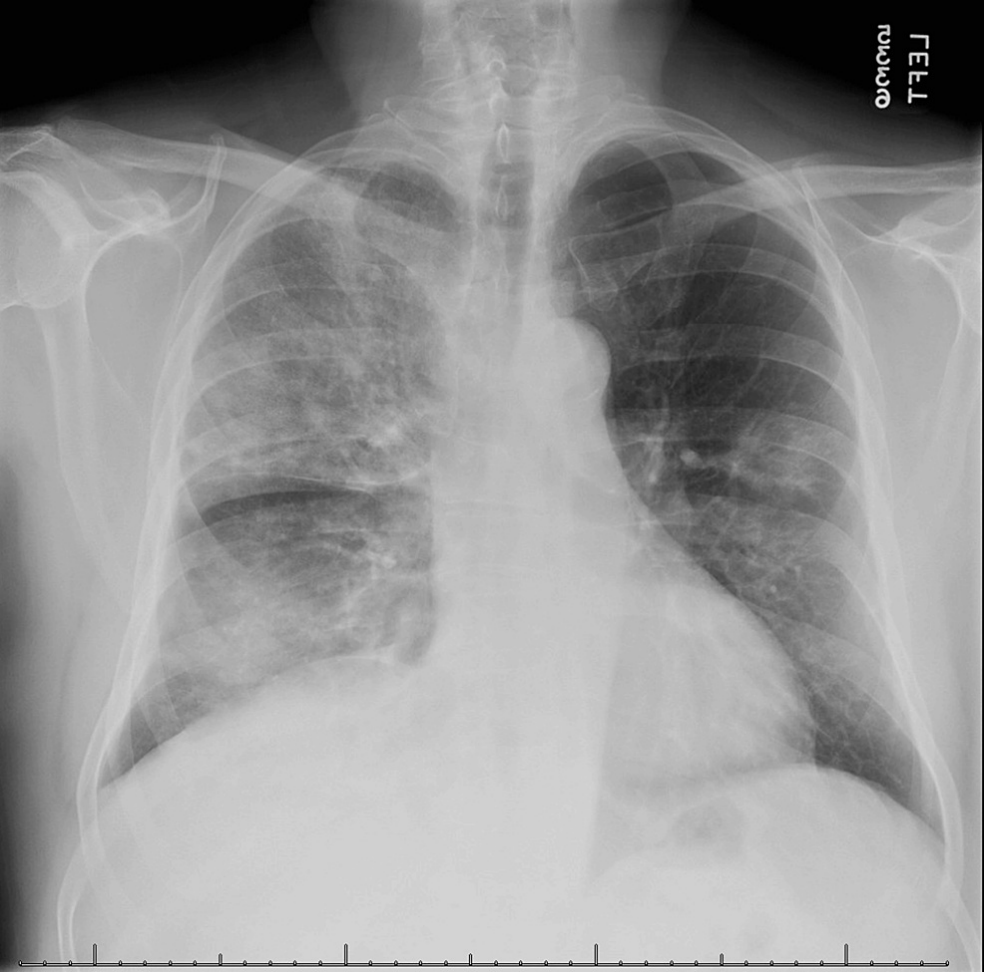
Patient's chest X-ray at the time of admission

Our patient is a 69-year-old man with a history of essential hypertension, Hepatitis C status post-treatment, and oral squamous cell carcinoma status postresection, who presented to the University of Texas Medical Branch (UTMB) Galveston emergency department with a chief complaint of a 10-day history of progressive fever and shortness of breath (SOB). This patient was initially seen at an outside clinic 10 days prior to presentation and was initially diagnosed with an upper respiratory infection (URI) and instructed to take ibuprofen and rest. Four days after his initial presentation, the patient developed nausea and diarrhea with progressively worsening fever and SOB. The patient and his wife presented at an urgent care due to worsening symptoms and were instructed to come directly to the emergency department. On admission, vitals were significant for a temperature of 101.8 °F, an oxygen saturation of 91%, pulse of 79 beats per minute, respiratory rate of 18, and blood pressure of 126/71 mm Hg. A respiratory exam demonstrated diffuse, bilateral crackles. The cardiovascular, abdominal, and bilateral upper and lower extremities examinations revealed no abnormalities. Labs were pertinent for the findings in Table 1 below, and a chest X-ray revealed diffuse involvement of both lungs with alveolar infiltrates, with the right greater than the left, consistent with multifocal pneumonia (Figure 1).

**Table 1:** Patient's pertinent lab abnormalities at the time of admission

Test Name	Patient's Result	Reference Range
Creatinine	3.3	0.6-1.2 mg/dL
Sodium	134	136-146 mEq/L
Aspartate aminotransferase (AST)	141	12-38 U/L
Alanine aminotransferase (ALT)	117	10-40 U/L

**Figure 2:**
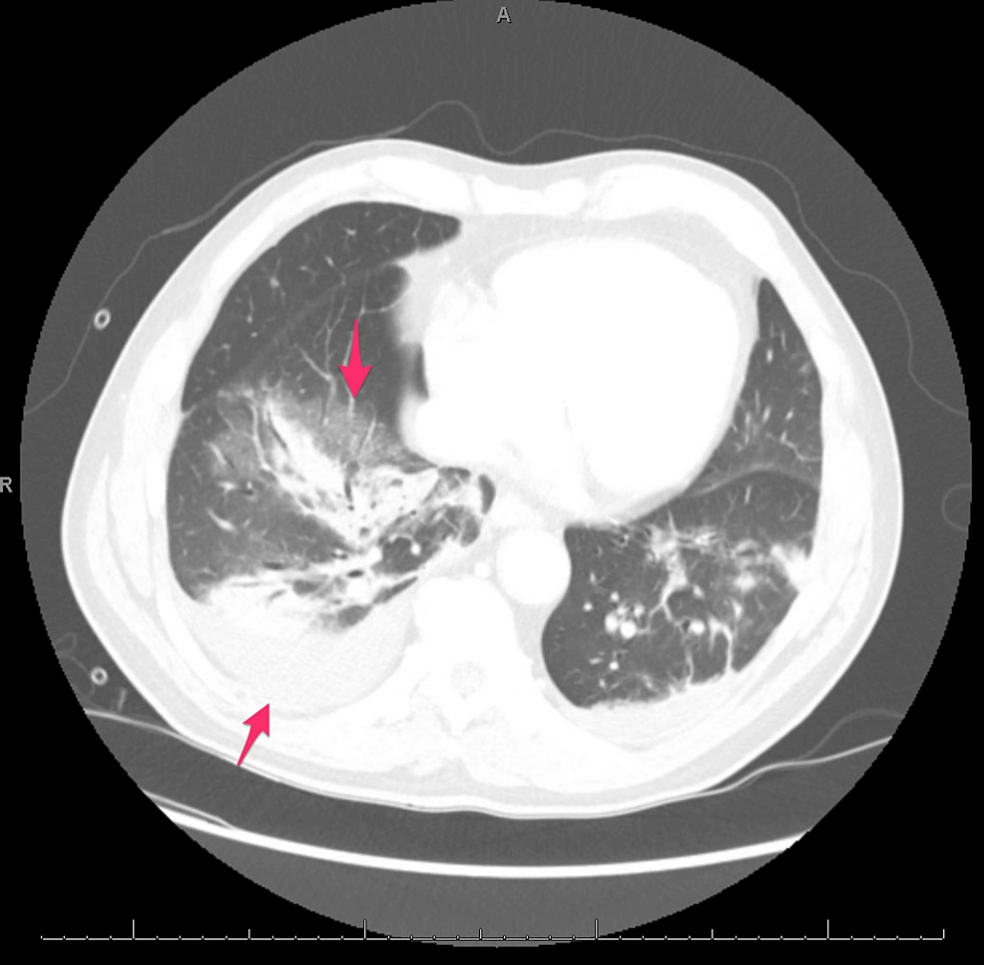
Patient’s CT chest with contrast The image is demonstrating multifocal opacities (top arrow) and parapneumonic effusion (bottom arrow).

Infectious workups, including coronavirus disease 2019 (COVID-19), Flu, and respiratory syncytial virus (RSV), were all negative. Urine samples for *Streptococcus pneumoniae* and *Legionella pneumophila* antigen testing were collected. The patient was admitted to the general medicine floor with a CURB-65 score of 2 and started on empiric antibiotics (intravenous (IV) rocephin, per os (PO) azithromycin). Following admission, the patient was persistently febrile and reported continued SOB with continued oxygen dependence of three liters via nasal cannula. Attempts at weaning oxygen use resulted in desaturations to 89%. Blood cultures showed no growth on hospital day three, and no microbial source had been identified. Perplexed as to why the patient showed no meaningful signs of improvement, we decided that further imaging was indicated with computed tomography (CT) chest with contrast. This patient had an elevated creatinine level of 1.94 at the time, so he was given pre-procedure hydration with intravenous fluids before imaging. The CT scan showed multifocal consolidative ground glass opacities, similar to prior, and a small parapneumonic effusion, which had increased in size from prior imaging (Figure 2).

Following the CT scan, we broadened antibiotic coverage with cefepime. On hospital day four, the patient's family reported that, two weeks ago, the patient had been removing standing water from his attic as a result of a local storm. It was only after discovering this additional information that the Legionella urine sample sent to an outside lab returned positive for Legionella pneumonia. Antibiotic coverage was adjusted to levofloxacin given minimal improvement with azithromycin previously, with subsequent satisfactory response to treatment. His oxygen requirements improved with time, and creatinine trended down and stabilized at around 1.80. He was afebrile for over 48 hours prior to discharge. The patient was discharged with instructions to continue levofloxacin 750 mg every 48 hours (renally dosed) to complete a 10-day total course. His creatinine continued to downtrend to 1.20 at outpatient follow-up one month after discharge.

## Discussion

Diagnosing Legionnaires’ disease requires a high degree of suspicion, as the disease closely resembles other forms of pneumonia both clinically and radiographically. Patients presenting with symptoms consistent with community-acquired pneumonia require a broad approach to management, as identification of the culprit organism is not immediately available. Our patient was appropriately tested for both typical and atypical organisms and started on appropriately broad antibiotic coverage. While Legionella remains relatively rare, with annual incidence in the USA being approximately 1.4 to 1.8 per 100,000 persons, its severity necessitates properly ruling it out of the differential [5]. As a reflection of its severity, Legionnaires’ disease has a mortality rate of 10% in patients without high-risk factors [1,6]. Recall that our patient had already been started on an appropriate agent on admission, a macrolide, for the appropriate duration for coverage of community-acquired pneumonia. However, given the patient’s lack of clinical improvement, our patient had potentially not been on the appropriate therapy for the required duration, as current literature reports reduced risk of mortality in patients with severe Legionnaires’ disease treated with a fluoroquinolone-based therapy [7].

An additional note in this patient’s hospital course was the use of a CT chest with contrast in the setting of elevated creatinine. While the incidence of contrast-induced nephropathy (CIN) is controversial, it was decided to use reno-protective measures of IV hydration. Reno-protective measures in and of themself are controversial, with one 2007 randomized controlled trial stating the superiority of hydration with sodium bicarbonate over normal saline alone and in conjunction with N-acetylcysteine [8], while a 2017 randomized controlled trial concluded no significant benefit of IV sodium bicarbonate over IV sodium chloride or of oral acetylcysteine over placebo [9]. We concluded that the clinical benefits of the diagnostic capability of CT with contrast would outweigh the risks associated with CIN and decided to proceed with imaging.

A final point worth noting is the delayed results of the urine antigen test. The literature reports that results are typically available within minutes to hours [6]. Diagnosis of Legionella pneumonia is most often made with urine antigen testing (UAT) and may also be made using sputum polymerase chain reaction (PCR), but the gold standard of detection is sputum culture [10]. However, as less than ½ of Legionella patients produce sputum (as seen in our patient with nonproductive cough), sputum for PCR may be challenging to obtain without using invasive methods such as bronchoalveolar lavage. UAT has high sensitivity and specificity (87% and 97.7%, respectively, when compared to sputum culture) and is a highly reliable method for detecting *L. pneumophila *serogroup 1, the cause of approximately 90% of Legionella pneumonia cases [10]. However, most commercially available UATs only detect this specific species and serotype, so Legionella pneumonia due to another variant may not be ruled out with a negative UAT. We believe that we would have ultimately arrived at the correct diagnosis regardless of the patient’s history of a possible contaminated water source. However, if the antigen testing had been in-house or had resulted earlier in his disease course, we may have been able to appropriately tailor the proper antibiotic course and reduce the patient’s time in the hospital.

## Conclusions

Suspicion for Legionnaires’ disease should be especially high if patients show any signs of atypical presentation, if gastrointestinal involvement is present, or if stereotypical laboratory abnormalities arise. Additionally, constant clinical re-evaluation is imperative, particularly for patients who do not display a meaningful response to appropriate therapy. Legionella pneumonia is a severe form of pneumonia that may progress rapidly if misdiagnosed or if treatment is delayed. Therefore, constant reassessment of clinical status is necessary, with repeat imaging as needed. Finally, consideration for alternative diagnoses is especially essential to ensure timely and appropriate management and, ultimately, better patient outcomes.
